# Analysis of cerebrospinal fluid chimerism after hematopoietic stem cell transplantation: Case report

**DOI:** 10.1016/j.htct.2024.04.129

**Published:** 2024-09-11

**Authors:** Yeliz Ogret, Tarik Onur Tiryaki, Cigdem Kekik Cinar, Ipek Yonal Hindilerden, Dilek Ozden Ozluk, Meliha Nalcaci, Fatma Savran Oguz

**Affiliations:** a Istanbul University Istanbul Faculty of Medicine Tissue Typing Laboratory, Turkey; b Istanbul University, Istanbul Faculty of Medicine, Department of Internal Medicine, Division of Hematology, Turkey

## Introduction

Allogeneic hematopoietic stem cell transplantation (allo-HSCT) is a successful treatment option in hematological malignancies. Treatment is followed by chimerism analysis, which is a method for determining whether the recipient's hematopoietic system is being rebuilt by donor cells.^1^^,^^2^ Central nervous system (CNS) complications have been described in 8–42% of patients undergoing allogeneic hematopoietic cell transplantation (allo-HCT). Diagnostic cerebrospinal fluid (CSF) analysis can be used in patients with neurological symptoms after allo-HCT.^3^

### Case

A 34-year-old man, who had a diagnosis of high-risk B-cell acute lymphoblastic leukemia (ALL), was in complete remission after BFM90 (prednisone, vincristine, asparaginase, cyclophosphamide, cytarabine, daunorubicin, doxorubicin, methotrexate and 6-mercaptopurine) plus craniospinal irradiation. After a myeloablative priming regimen of busulfan plus cyclophosphamide, Allo-HSCT was performed in 2019 with a donation from his 26-year-old nulliparous sister, who had fully compatible HLA. Steroid, rituximab and ruxolitinib treatments were administered for severe chronic graft-versus-host disease (GvHD) with skin, liver and muscle-nerve involvement that started during the tapering period of immunosuppressive therapy three months after transplantation. Partial response was obtained of the chronic GvHD with these treatments. Cranial imaging, which revealed no pathology, was performed due to facial numbness in the 26th month after transplantation. CNS recurrence was observed in a cytological examination of cerebrospinal fluid. No sign of disease was observed in a systemic evaluation: bone marrow and peripheral blood analysis showed complete chimerism. Loss of chimerism was detected in a CSF sample of the patient, characterized as isolated CNS relapse, and so contributed to the diagnosis of relapse. An increase in CSF chimerism was seen in favor of the donor with three courses of systemic HyperCVAD (cyclophosphamide, vincristine sulfate, doxorubicin hydrochloride, and dexamethasone) treatment. Additionally, intrathecal treatments (40 mg cytosine arabinoside, 12 mg methotrexate and 8 mg dexamethasone) were administered twice a week until no cells were detected in the CSF and then once a week for four weeks. Transplantation was performed in the second complete remission after a total body irradiation-based priming regimen using a donation from another HLA-matched sibling. Remission followed in the 8th month after transplantation.

Short tandem repeat (STR) chimerism analysis of the bone marrow and peripheral blood from the 1st month after transplantation was found to have complete chimerism (Table 1). Although the peripheral blood chimerism study performed in the 26th month after the transplantation showed complete chimerism, the chimerism analysis performed using a DNA sample obtained from the CSF on the same day due to numbness of the face was found to have recipient-derived STR alleles. The cytological analysis of CSF also confirmed the recurrence in the CNS. After ALL and CNS intrathecal treatments, no recurrence was observed in the pathological examination of CSF, however, mixed chimerism with donor cells and clinical relapse were detected (neurological complaints regressed and CSF cell count became negative) in the chimerism analysis performed with CSF at Month 34.

**Table 1:** Patient short tandem repeat analysis results.

Month after transplant	Sample Type	Chimerism Status	Clinical Status
1st	BM	CC	GvHD
	PB	CC	
6th	PB	CC	cGvHD
21st	BM	CC	
	PB	CC	
26th	PB	CC	CNS involvement
	CSF	<5% donor cells	
29th	BM	CC	
	PB	CC	
32nd	BM	CC	CNS relapse
	PB	CC	
33rd	BM	CC	CNS relapse
	PB	CC	
34th	CSF	MC (23% donor cells)	CNS relapse

BM: Bone marrow; PB: Peripheral blood; CC: Complete chimerism; MC: Mixed chimerism; GvHD: Graft-versus-host disease; CNS: Central nervous system.

## Discussion and conclusion

Determination of cell count, evaluation of cell morphology and flow cytometric analysis of CSF cells are useful as complementary techniques for examining CNS recurrence in leukemia and lymphoma.^1^^,^^2^ Donor chimerism status is tested both in peripheral blood and bone marrow using STR analysis, however, very few articles using STR analysis of CSF have been published. In the only published study, Waterhouse et al. recently reported the clinical benefits of CSF STR analysis in adult patients presenting with neurological complications at different times following allo-HSCT.^2^ The authors showed that a moderate correlation was found between the percentage of recipient DNA determined by peripheral blood STR amplification and the cell concentration in CSF.^2^ In the current study, although STR analysis of bone marrow and peripheral blood showed complete chimerism, loss of chimerism was found in CSF. We think that it would be more sensible to perform the chimerism STR analysis with CSF instead of peripheral blood or bone marrow in the follow-up of HSCT in patients with neurological findings. As a matter of fact, the mixed chimerism result obtained from the patient who was treated after loss of CSF chimerism shows that it plays an important role in the early diagnosis and follow-up of the treatment of CNS recurrence. Analysis of donor-recipient chimerism in CSF in patients with neurological symptoms after allo-HSCT is a practical, feasible and useful method.

## Conflicts of interest

The authors declare no conflicts of interest.

## References

[1] Spruit J.L., Fagoaga O.R., Savaşan S. (2018). Donor cell chimerism evaluation in cerebrospinal fluid by short tandem repeat analysis following allogeneic hematopoietic stem cell transplantation. Hum Immunol.

[2] Lindahl H., Valentini D., Vonlanthen S., Sundin M., Björklund A.T., Mielke S. (2022). Early relapse prediction after allogeneic hematopoietic stem cell transplantation for acute lymphoblastic leukemia (ALL) using lineage-specific chimerism analysis. EJHaem.

[3] Waterhouse M., Bartsch I., Bertz H., Duyster J., Finke J. (2016). Cerebrospinal fluid chimerism analysis in patients with neurological symptoms after allogeneic cell transplantation. Bone Marrow Transplant.

